# The role of RNA methylation in tumor immunity and its potential in immunotherapy

**DOI:** 10.1186/s12943-024-02041-8

**Published:** 2024-06-20

**Authors:** Yan Li, Haoer Jin, Qingling Li, Liangrong Shi, Yitao Mao, Luqing Zhao

**Affiliations:** 1 Department of Pathology, Xiangya Hospital, Central South University, Changsha, Hunan China; 2 Department of Pathology, School of Basic Medical Science, Xiangya School of Medicine, Central South University, Changsha, Hunan China; 3 Department of Radiology, Xiangya Hospital, Central South University, Changsha, Hunan China; 4 National Clinical Research Center for Geriatric Disorders, Xiangya Hospital, Central South University, Changsha, Hunan China

**Keywords:** RNA methylation, Tumor immunity, Immunotherapy, Tumor immune evasion, Tumor microenvironment (TME)

## Abstract

RNA methylation, a prevalent post-transcriptional modification, has garnered considerable attention in research circles. It exerts regulatory control over diverse biological functions by modulating RNA splicing, translation, transport, and stability. Notably, studies have illuminated the substantial impact of RNA methylation on tumor immunity. The primary types of RNA methylation encompass N6-methyladenosine (m6A), 5-methylcytosine (m5C), N1-methyladenosine (m1A), and N7-methylguanosine (m7G), and 3-methylcytidine (m3C). Compelling evidence underscores the involvement of RNA methylation in regulating the tumor microenvironment (TME). By affecting RNA translation and stability through the "writers", "erasers" and "readers", RNA methylation exerts influence over the dysregulation of immune cells and immune factors. Consequently, RNA methylation plays a pivotal role in modulating tumor immunity and mediating various biological behaviors, encompassing proliferation, invasion, metastasis, etc. In this review, we discussed the mechanisms and functions of several RNA methylations, providing a comprehensive overview of their biological roles and underlying mechanisms within the tumor microenvironment and among immunocytes. By exploring how these RNA modifications mediate tumor immune evasion, we also examine their potential applications in immunotherapy. This review aims to provide novel insights and strategies for identifying novel targets in RNA methylation and advancing cancer immunotherapy efficacy.

## Introduction

**Fig. 1:**
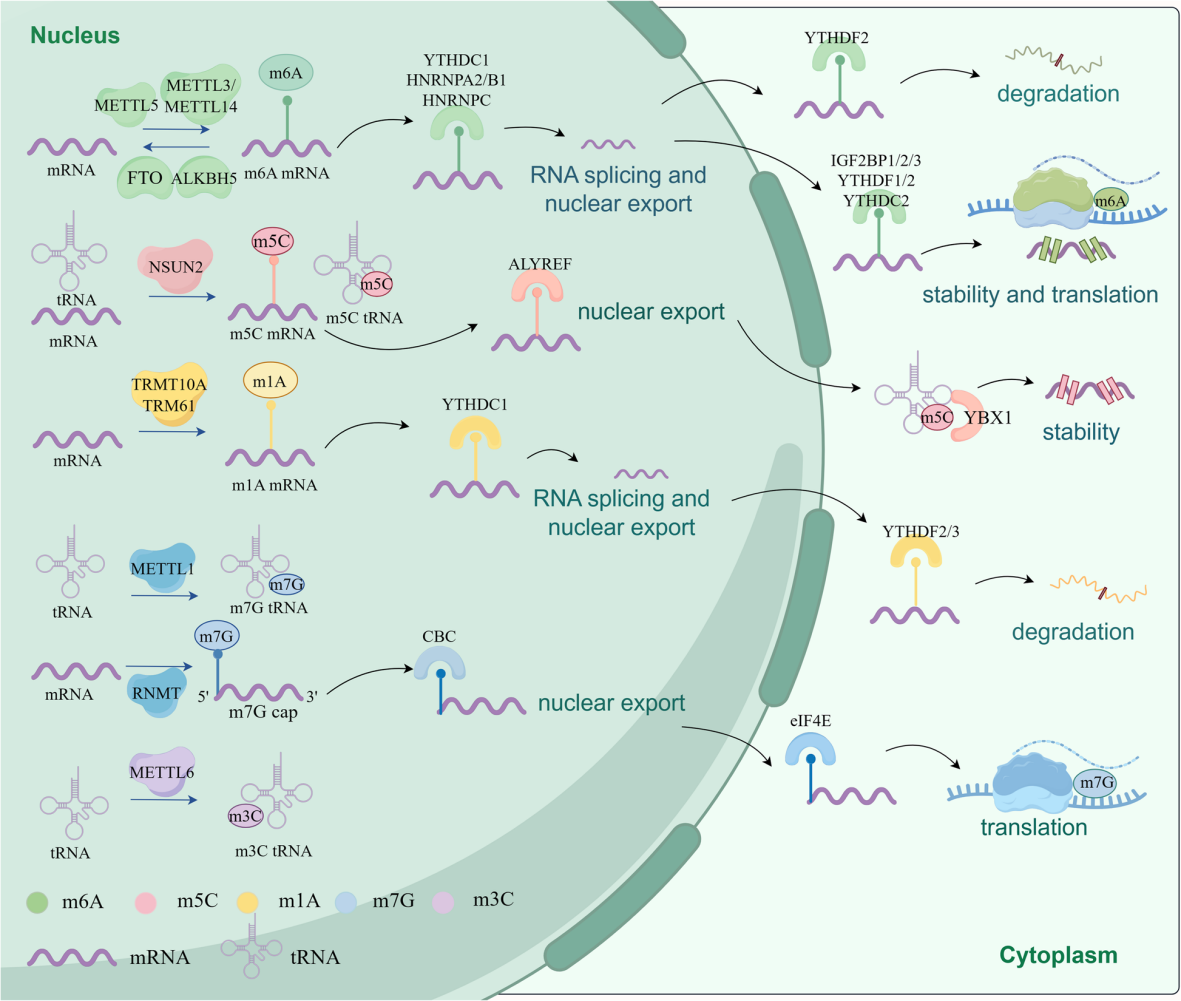
The machinery of RNA methylations and RNA fates regulated by RNA methylations. RNA methylations are modulated by their writers (such as METTL3/14 for m6A, NSUN2 for m5C, TRMT10A for m1A, METTL1 for m7G), and removed by their erasers (such as FTO and ALKBH5 for m6A). RNA methylations can regulate the fates of mRNA and mediate their biological functions including splicing, exportation, stability, degradation, translation and so on, after being recognized by their respective readers, including IGF2BP1/2/3, YTHDF1/2/3, YTHDC1/2/3, YBX1, ALYREF, CBC, eIF4E). *m6A* N6-methyladenosine, *m5C* 5-methylcytosine, *m1A* N1-methyladenosine, *m7G* 7-methylguanosine, *m3C* 3-Methylcytidine, *METTL3* methyltransferase-like 3, *FTO* obesity-associated protein, *ALKBH5* AlkB homolog 5, *TET1/2/3* ten-eleven translocation proteins1/2/3, *ALKBH1* α-ketoglutarate-dependent dioxygenase ABH1. Figure created with figdraw.com

RNA modification critically influences gene expression through chemical changes to RNA bases and ribose. To date, researchers have identified over 170 types of chemical modifications in various RNA classes across both prokaryotes and eukaryotes [1, 2]. Among these, RNA methylation, which accounts for more than 60% of all RNA modifications, plays a pivotal role in post-transcriptional gene regulation [1, 3, 4]. The major forms of RNA methylation include N1-methyladenosine (m1A), N6-methyladenosine (m6A), 5-methylcytosine (m5C), N7-methylguanosine (m7G), and 3-methylcytidine (m3C), highlighting its extensive presence and significance in shaping the complex landscape of gene regulation [1, 2, 5, 6]. RNA methylation is mediated by three types of proteins: "writers," which catalyze the addition of methyl groups; "readers," which identify these modifications; and "erasers," which remove them, each functioning through unique mechanisms [2, 5, 7] (Fig. 1). These proteins regulate a wide array of RNA types and signaling pathways, including mRNA, tRNA, IncRNA, sRNA, siRNA, snRNA, snoRNA, etc. As a dynamic and reversible process, RNA methylation regulates critical biological processes such as splicing, translation, transport, and RNA stability. Extensive studies have demonstrated that RNA methylation is crucial in the development and progression of various types of cancer, including breast cancer, lung cancer, colorectal cancer (CRC), hepatocellular carcinoma (HCC), gastric cancer (GC), esophageal cancer (EC), prostate cancer (PCa), bladder cancer, ovarian cancer, acute myeloid leukemia (AML), pancreatic cancer, etc. [1, 4, 8–16], underscoring its key role in malignant tumors.

In recent years, numerous studies have underscored the close association between RNA methylation and various immune biological processes, particularly within the context of tumor immunity [17, 18]. Additionally, abnormal expression of regulatory proteins has been linked to oncogenic activities and enhanced metastatic properties [19]. RNA methylation also plays a crucial role in maintaining homeostasis and in the metabolic reprogramming of the tumor microenvironment (TME), impacting the functionality of immune cells. The TME consists of a complex multicellular matrix that includes immune cells, stromal cells, the extracellular matrix, blood vessels, and other soluble factors [20]. RNA methylation contributes to tumor immune evasion by influencing oncogenic and metastatic capabilities, disrupting TME harmony, and impairing immune cell function. For instance, the m6A writer METTL3 is known to sustain high levels of glycolysis and to induce metabolic reprogramming in HCC [21]. This enzyme also affects macrophage polarization, dendritic cell activation, effector T cell differentiation and proliferation, and the expression of immune checkpoints [22–25]. These interactions highlight how RNA methylation connects the TME and immune cells with the mechanisms of tumor immune evasion. Currently, researchers are exploring potential inhibitors that target METTL3 and other RNA methylation regulators, with the hope that these compounds might be utilized in immunotherapy [26].

Components of the TME exhibit either anti-tumor or pro-tumor properties and play crucial roles in the initiation, progression, invasion, and metastasis of tumors. RNA methylation influences the biological processes of immune cells and other cellular components within the TME. Research has demonstrated that targeting these regulatory proteins can significantly advance cancer immunotherapy [11]. Immunotherapy seeks to boost anti-tumor immune responses by modulating the immune cells of the host's immune system, thereby aiding in the elimination of tumor cells. Focusing on the immune infiltrates within the TME has emerged as a promising approach that can decisively improve the clinical outcomes for cancer patients [27].

RNA methylation significantly influences cellular metabolism and plays a regulatory role in TME and immune cells, crucially impacting tumor immunity. Importantly, it is involved in the development and progression of various human diseases, including AML, CRC, GC, glioblastoma (GBM), renal cell carcinoma (RCC), HCC, etc. [28–31]. This paper will comprehensively explore the role of RNA methylation in tumor immunity and its potential applications in immunotherapy. Our discussion aims to offer new insights and strategies for the development of innovative targets for cancer diagnosis, treatment, and prognosis.

## Classification of RNA methylation

### N6-methyladenosine

N6-Methyladenosine (m6A), the predominant form of methylation in human mRNA, modifies adenosine at the N6 position and constitutes about 60% of RNA methylation events [4, 8, 32]. This modification is not only prevalent in mammalian mRNA but also occurs across a wide range of non-coding RNAs, including ribosomal RNAs (rRNAs), microRNAs (miRNAs), small nuclear RNAs (snRNAs), small nucleolar RNAs (snoRNAs), long non-coding RNAs (lncRNAs), and circular RNAs (circRNAs) [7, 32–34]. m6A critically influences RNA stability, transport, splicing, and translation, thereby affecting overall RNA expression [2, 8, 32]. The dynamic regulation of m6A involves various components such as methyltransferases (writers), demethylases (erasers), and methylation reading proteins (readers). The m6A methyltransferase complex (MTC), which includes METTL3, METTL14, WTAP, RBM15/15B, ZC3H13, VIRMA, and KIAA1429, plays a vital role in catalyzing m6A modification on different RNA types [25, 35, 36]. The demethylation process is controlled by demethylases like FTO and ALKHB5, although METTL5, responsible for 18S rRNA m6A modification, currently has no known erasers or readers [37]. m6A methylation reader proteins encompass a diverse array of molecules, including insulin-like growth factor 2 mRNA-binding proteins 1/2/3 (IGF2BP1/2/3), YTH domain family proteins 1/2/3 (YTHDF1/2/3), embryonic Lethal Abnormal Vision Like 1 (ELAVL1), eukaryotic translation initiation factors 3 (eIF3), 4E (eIF4E), and 4G (eIF4G), poly(A) binding protein (PABP), etc. [38, 39]. These reader proteins possess the ability to recognize bases bearing m6A modifications, thereby initiating a cascade of downstream effects including translation, splicing, nuclear exportation, and degradation [38, 39] (Fig. 1). Moreover, they can specifically bind to m6A sites on RNA, thereby influencing disease onset and progression by modulating RNA stability and translation. For instance, IGF2BP3 has been implicated in promoting tumorigenesis and predicting poor prognosis in AML through its enhancement of regulator of chromosome condensation 2 (RCC2) stability [40]. Similarly, YTHDF1 has been shown to drive ovarian cancer progression by facilitating EIF3C translation [41]. Numerous studies have highlighted the involvement of m6A regulators in a wide range of human diseases, spanning psychiatric disorders, metabolic diseases, cardiovascular diseases, as well as specific cancers such as AML, brain tumors, bladder cancer, ovarian cancer, etc. [39–45] (Table 1).

**Table 1:** The regulator proteins of RNA methylations

Methylations	Regulator	Molecular	Cancer type	Biological function	References
m6A	Writers	METTL3	CRC	Promote oncogenesis via GLUT1 translation	[46]
			AML	Promote oncogenesis via ITGA4 stability	[44]
			Endometrial cancer	Inhibit oncogenesis via NLRC5 degradation	[47]
			GC	Promote oncogenesis via HDGF stability	[48]
		METTL14	HCC	Promote oncogenesis via SIRT6 stability	[49]
		METTL16	CRC	Promote oncogenesis via PD-L1 translation	[50]
		WTAP	HCC	Promote oncogenesis via HuR translation	[51]
		ZC3H13	CRC	Inhibit oncogenesis via snail and cyclin D1 translation	[52]
			HCC	Inhibit oncogenesis via PKM2 stability	[53]
		VIRMA	NSCLC	Promote oncogenesis via DAPK3 degradation	[54]
		KIAA1429	DLBCL	Promote oncogenesis via CHST11 translation	[55]
	Erasers	FTO	CRC	Inhibit oncogenesis via PD-L1 translation	[56]
			Melanoma	Promote oncogenesis via PDCD1 translation	[57]
		ALKBH5	CRC	Promote oncogenesis via AXIN2 stability	[58]
			HCC	Promote oncogenesis via MAP3K8 translation	[59]
				Promote oncogenesis via GLUT4 mRNA stability	[60]
	Readers	IGF2BP3	AML	Promote oncogenesis via RCC2 stability	[40]
			HCC	Promote oncogenesis via CCL5 translation	[61]
		YTHDF1	NSCLC	Promote oncogenesis via cyclin D1 translation	[62]
		ELAVL1	MPNSTs	Promote oncogenesis via HuR translation	[63]
m5C	Writers	DNMT2	AML	Promote oncogenesis via hnRNPK translation	[45]
		NOP2	HCC	Promote oncogenesis via c-Myc translation	[12]
		NSUN2	EC	Promote oncogenesis via GRB2 stability	[10]
			GC	Promote oncogenesis via PIK3R1 translation	[9]
		NSUN6	Lung cancer	Promote oncogenesis via NM23-H1 translation	[11]
		NSUN7	HCC	Promote oncogenesis via CCDC9B stability	[12]
	Erasers	ALKBH1	CRC	Promote metastasis via SMAD7 translation	[64]
	Readers	ALYREF	Bladder cancer	Promote oncogenesis via PKM2 stability	[53]
		YBX1	AML	Promote oncogenesis via BCL2 stability	[65]
m1A	Writers	TRMT61A	HCC	Promote oncogenesis via PPARδ translation	[13]
		TRM6	GC	Promote oncogenesis via ErbB translation	[14]
	Erasers	ALKBH1	Pancreatic cancer	Promote oncogenesis via mTOR and ErbB translation	[15]
		ALKBH3	Breast cancer	Promote oncogenesis via CSF-1 mRNA stability	[66]
			Ovarian cancer	Promote oncogenesis via CSF-1 mRNA stability	[66]
			Cervical cancer	Promote oncogenesis via ATP5D mRNA translation	[67]
	Readers	YTHDF3	Cervical cancer	Promote oncogenesis via ATP5D mRNA translation	[67]
		YTHDC1	PDAC	Inhibit oncogenesis via miR-30d mRNA stability	[68]
m7G	Writers	METTL1	ACC	Promote oncogenesis via HK1 translation	[69]
			HCC	Promote oncogenesis via Cyclin A2 and EGFR translation	[70]
			HCC	Promote oncogenesis via TGF‐β2 mRNA translation	[71]
			Bladder cancer	Promote oncogenesis via EGFR/EFEMP1 mRNA translation	[72]
		WDR4	HCC	Promote oncogenesis via Cyclin A2 and EGFR mRNA translation	[70]
		RNMT	Gliomas	Promote oncogenesis via c-Myc mRNA translation	[73]
			Breast cancer	Promote oncogenesis via PIK3CA translation	[74]
		WBSCR22	Pancreatic cancer	Inhibit oncogenesis via ISG15 translation	[16]
		TRMT112	Pancreatic cancer	Inhibit oncogenesis via ISG15 translation	[16]
m3C	Writer	METTL6	HCC	Promote oncogenesis	[75]

*Abbreviation*: *ACC* Adrenocortical carcinoma, *AML* Acute myeloid leukemia, *CRC* Colorectal cancer, *DLBCL* Diffuse large B-cell lymphoma, *EC* Esophageal cancer, *GC* Gastric cancer, *HCC* Hepatocellular carcinoma, *MPNSTs* Malignant peripheral nerve sheath tumors, *NSCLC* Non-small cell lung cancer, *PDAC* Pancreatic ductal adenocarcinoma

### 5-methylcytosine

5-Methylcytosine (m5C) is a chemical modification found at the fifth carbon atom of cytosine in RNA molecules. This modification is extensively distributed across various RNA types, including transfer RNA (tRNA), ribosomal RNA (rRNA), messenger RNA (mRNA), non-coding RNA (ncRNA), enhancer RNA (eRNA), and microRNA (miRNA) [76, 77]. Despite its discovery over fifty years ago, the specific functions of m5C are still not fully elucidated [78]. RNA bisulfite sequencing, the most commonly used method to map m5C locations, has shown that these sites are predominantly enriched in the 3′-untranslated regions (3′-UTR) of mRNAs or near the translation initiation codon [79]. m5C plays multiple crucial roles in RNA biology: it enhances mRNA stability and structure, ensures translation accuracy, maintains integrity of tRNA fragments, influences the translation of stop codons in rRNA, and regulates the nuclear export of mature mRNAs [79–83] (Fig. 1).

m5C significantly impacts various biological processes including cell proliferation, differentiation, migration, and apoptosis [84, 85]. The enzymatic addition of m5C is facilitated by "writers" such as DNA methyltransferase 2 (DNMT2) and members of the NOP2/SUN RNA methyltransferase family, including NSUN1 through NSUN7 [85–87]. NSUN2, 3, 6, and DNMT2 have all been demonstrated to methylate tRNAs. Notably, NSUN2 is an essential RNA methyltransferase responsible for introducing m5C to RNA. It methylates most expressed tRNAs, along with other abundant non-coding RNAs and a few of mRNAs [82, 88, 89]. The cancer stem cell functions are controlled by global protein synthesis, but NSUN2 depletion induces decreased m5C level of tRNA and inhibits this process [83]. In budding yeast, NOP2/NSUN1 is essential for ribosome biogenesis, as it deposits m5C on 25S rRNA [90]. NSUN5 modulates protein synthesis by targeting m5C on 28S rRNA [91], while NSUN6 is crucial in regulating cell proliferation in pancreatic cancer and may serve as a potential biomarker for this disease [92]. The removal of m5C is performed by "erasers" such as the Ten-eleven translocation (TET) proteins (TET1-3) and α-ketoglutarate-dependent dioxygenase ABH1 (ALKBH1), which can oxidize m5C to 5-hydroxymethylcytidine (hm5C) [93–95]. Meanwhile, m5C is regulated by its reader proteins, specifically Aly/REF export factor (ALYREF) in mRNA and Y-box-binding protein 1 (YBX1) in tRNA [96] (Fig. 1). Research has shown that ALYREF can directly recognize and bind to the m5C sites in mRNA to promote the export of mRNA from the nucleus to the cytoplasm [97]. YBX1 also binds m5C to regulate its presence in both coding and non-coding RNA and affects rRNA maturation [98, 99]. Additionally, YBX1 interacts with hsa_circ_0062682 to modulate RNA metabolism and splicing, promoting proliferation and invasion in HCC cells, and contributing to sorafenib resistance [100]. Despite the significant roles of these proteins, research into m5C readers for tRNA and rRNA is still in its infancy. ALYREF and YBX1 are linked to the progression of HCC and AML through their influence on BCL2 mRNA stability, suggesting their potential as indicators of poor prognosis and reduced survival [65, 101] (Table 1).

### N1-methyladenosine

First identified in the 1960s, N1-methyladenosine (m1A) results from the methylation of adenosine at position 1 and has been detected in tRNAs, rRNAs, mRNAs, and lncRNAs [102–104]. This reversible modification is catalyzed by several enzymes, including tRNA methyltransferase 10 homologue A (TRMT10A) at four specific positions and the TRM6–TRM61 complex, which targets mRNA and mitochondrial tRNA [105, 106]. Additional writers of m1A include nucleomethylin (NML, also known as RRP8) for rRNA, TRMT61A and TRMT61B for mitochondrial tRNA and rRNA, TRMT10B for tRNA, and TRMT10C for mitochondrial tRNA and mRNA [107, 108]. As a post-transcriptional modification, m1A significantly influences RNA stability by affecting base pairing [109]. The removal of m1A is facilitated by "erasers" such as FTO, ALKBH1, ALKBH3, ALKBH5, and ALKBH7, which demethylate various RNA types. Specifically, FTO, ALKBH1, and ALKBH7 target tRNA, whereas ALKBH3 is active on both tRNA and mRNA [57, 64, 110–112]. Although these m1A erasers share some functions with m6A erasers, the specific proteins that recognize m1A in RNA remain unidentified. However, several m6A readers, including YTHDF1/2/3 and YTHDC1, have been shown to detect m1A modifications and directly interact with them [113] (Fig. 1 and Table 1).

### N7-methylguanosine

N7-methylguanosine (m7G) is an RNA methylation modification occurring at the N7 position of guanine, accounting for approximately 0.4% of all guanosine residues [114]. This modification is typically found at the 5’ caps and internal sites of mRNA, as well as within rRNA, tRNA, and miRNA [115–117]. The primary enzyme responsible for this modification is methyltransferase-like 1 (METTL1), which partners with the WD repeat domain 4 (WDR4) complex to insert m7G modifications into tRNA, miRNA, and mRNA, thus influencing miRNA structure and biogenesis [118, 119]. Additionally, RNA guanine-7 methyltransferase (RNMT) and RNMT-activating miniprotein (RAM) play critical roles in the efficient cap methylation of mRNA by applying the m7G modification [73, 120]. Furthermore, Williams–Beuren syndrome chromosome region 22 (WBSCR22) and tRNA methyltransferase activator subunit 112 (TRMT112) also contribute to m7G methylation in rRNA [119, 121]. eIF4E is known to recognize the m7G cap of mRNA and plays a crucial role in mediating mRNA translation. Together with the cap-binding complex (CBC), which includes CBP80 and CBP20, it significantly influences the nuclear export and translation of mRNA [122–124] (Fig. 1). Extensive research has linked m7G methylation to various aspects of tumor biology such as stress responses, and the initiation, progression, and prognosis of cancer [125]. Notably, the m7G modification, catalyzed by METTL1 and WDR4 on tRNA, is markedly increased in cancer patients, affecting a range of malignancies including AML, HCC, prostate cancer (PCa), and bladder cancer [71, 72, 126–128]. Additionally, abnormal expression patterns of RNMT have been observed in breast cancer and gliomas, highlighting its potential involvement in tumorigenesis and disease progression [74, 129] (Table 1).

### 3-Methylcytidine

3-Methylcytidine (m3C) is a modification found specifically in eukaryotic tRNA [130]. This modification occurs at position 32 and plays a crucial role in determining the structure and function of tRNA. Current research suggests that m3C methylation might be catalyzed by specific methyltransferases, with studies pointing to METTL2A, METTL6, and METTL8 as key enzymes involved in this process [75, 130, 131]. However, the understanding of m3C methylation is still limited, and further studies are essential to elucidate the underlying mechanisms and identify the associated regulatory proteins.

### RNA Methylation Regulates Tumor Microenvironment (TME)

The tumor microenvironment (TME) comprises the surroundings of tumor cells, encompassing blood vessels, immunocytes, fibroblasts, cytokines, the extracellular matrix, and various stromal components [132, 133]. Immunological elements within the TME coordinate tumor immunity [134–136]. TME significantly influences tumor initiation, progression, metastasis, and response to treatment [134, 137].

**Fig. 2:**
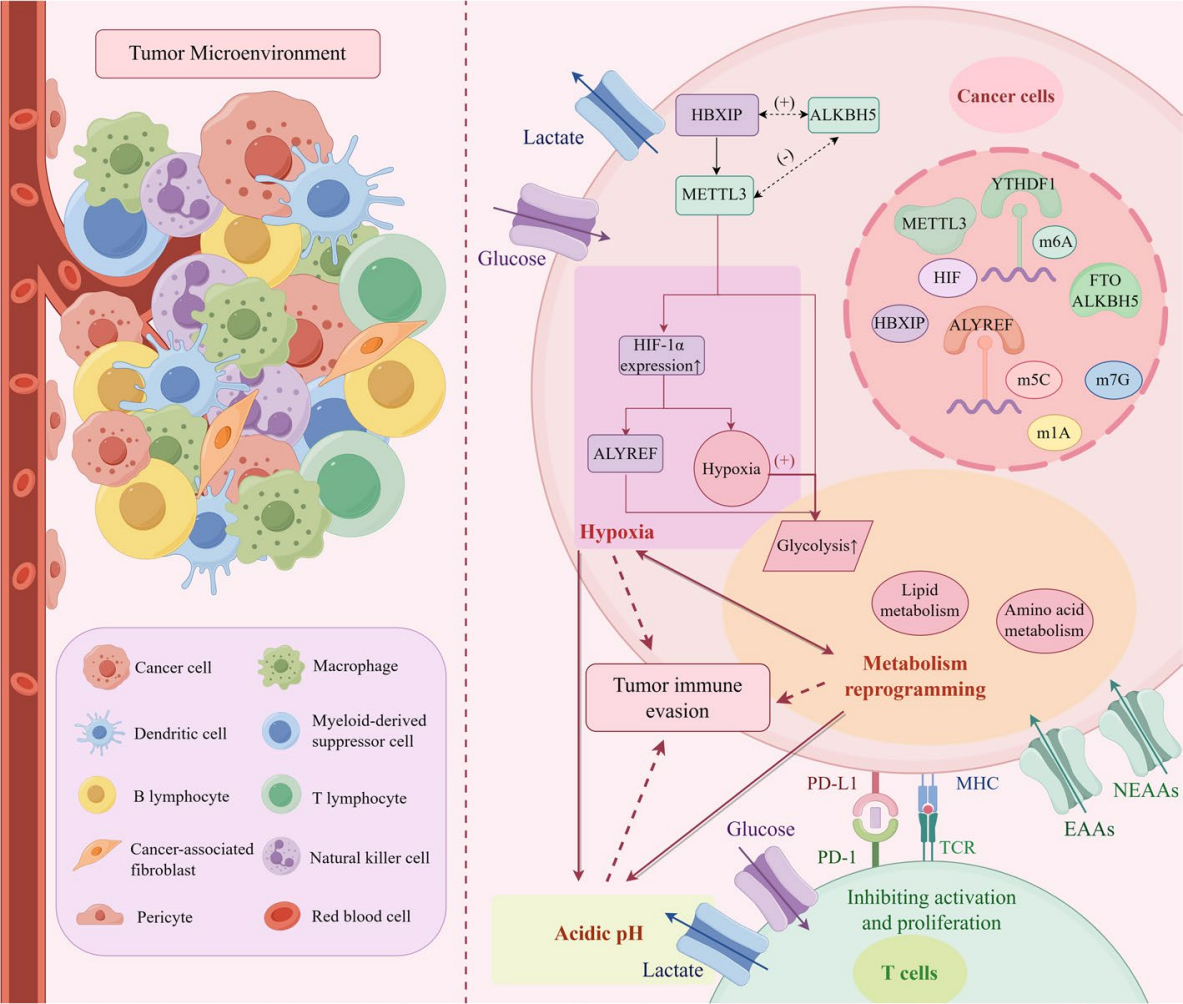
The compositions of tumor microenvironment (TME) and RNA methylations promote tumor immune evasion through hypoxia, metabolic reprogramming and acidic pH environment. Hypoxia-inducible factor (HIF) regulates the formation of immunosuppressive TME and promotes tumor immune escape by m6A, m5C, m1A, and m7G RNA methylations. RNA methylations regulate biological metabolism, including glucose metabolism, lipid metabolism and amino acid metabolism, leading to immune cell dysfunction and the formation of an acidic environment, which promotes tumorigenesis, angiogenesis, and tumor cell proliferation. This further aggravates tissue hypoxia and promotes tumor progression. Hypoxia, metabolic reprogramming, and acidic environment interact with each other and work together to contribute to tumor immune escape. TME, tumor microenvironment; HIF, hypoxia-inducible factors. NEAAs, non-essential amino acids; EAAs, essential amino acids. Figure created with figdraw.com

RNA methylation plays a pivotal role in shaping the complexity and diversity of the TME, exerting regulatory control over the initiation, progression, and metastasis of various cancers, including HCC, PCa, GC, CRC, pancreatic ductal adenocarcinoma (PDAC), non-small cell lung cancer (NSCLC), small-cell lung cancer (SCLC), malignant peripheral nerve sheath tumors (MPNSTs), etc. [33, 51–54, 63, 138] (Table 1). The m6A modification, a prominent form of RNA methylation, is implicated in a plethora of RNA biology processes, spanning RNA processing, translation, stabilization, splicing, and degradation. Consequently, it exerts influence over the dynamic landscape of the TME, impacting the metabolic and biological functions of tumor cells [138, 139]. Interactions between tumor cells and the TME significantly contribute to processes such as proliferation, differentiation, invasion, metastasis, and development of drug resistance [138]. The TME is typified by three key features: hypoxia, metabolic reprogramming, and immune evasion, which collectively foster the establishment of an immunosuppressive microenvironment and regulate tumor immune evasion through various mechanisms [28, 133, 140] (Fig. 2). Substantial evidence suggests that m6A methylation actively participates in tumor immune evasion by modulating the immunosuppressive TME [132, 141, 142]. Thus, we comprehensively explore the composition of the TME, elucidate the molecular mechanisms governing RNA methylation regulation, and delineate its role in mediating the biological effects of tumor immunosuppression (Fig. 2).

#### Hypoxic

Hypoxia stands out as a prominent feature within the tumor microenvironment, tightly interlinked with tumorigenesis, angiogenesis, metabolism, and immune response [143, 144]. Excessive hypoxia within tissues disrupts microenvironmental homeostasis, fostering the emergence of a hypoxic, hypoglycemic, and acidic TME conducive to tumor initiation and growth [145, 146]. The rapid proliferation of tumor cells exacerbates oxygen depletion within the tissue, exacerbating microenvironmental hypoxia. Hypoxia-inducible factors (HIF) play a pivotal role in activating genes associated with cellular oxygen homeostasis, including those involved in glucose and lactate metabolism. This activation favors glycolysis over oxidative metabolism, creating a conducive environment for tumor cell proliferation [142, 145–147]. HIF is intricately linked to tumor metabolism and plays a crucial role in immune evasion.

m6A methylation plays a pivotal role in shaping the hypoxic, hypoglycemic, and acidic tumor microenvironment, with the levels of its regulators closely linked to tumor cell content [20, 124, 127]. For instance, YTHDF1, an m6A reader protein, collaborates with other m6A-specific mRNA binding and translation proteins to regulate the methylation and expression of HIF genes, thereby promoting hypoxia-associated tumor progression [62]. Additionally, under hypoxic conditions, HBx-interacting protein (HBXIP) enhances METTL3 expression, a component of the m6A methyltransferase complex. This upregulation of METTL3 results in increased expression of HIF-1α and maintenance of elevated glycolysis levels, thereby accelerating the progression of HCC [21] (Fig. 2). METTL3 and its downstream reader YTHDF1 have been shown to participate in the upregulation of HIF expression and the acceleration of glycolysis [146, 148]. Furthermore, studies indicate that hypoxia suppresses FTO protein expression, correlating with a high recurrence rate and poor prognosis in patients with CRC [56]. Additionally, the overexpression of ALKBH5 promotes tumor progression by establishing a positive feedback loop with HBx protein. This loop leads to the upregulation of ALKBH5 via H3K4me3 epigenetic modification of the ALKBH5 promoter, resulting in the removal of m6A [149]. However, some investigations propose that METTL3 and ALKBH5 contribute to the establishment of opposing hypoxia and reoxygenation conditions, thereby regulating m6A methylation in ischemic heart disease [150]. Therefore, a coordinated interplay between m6A methylation and hypoxia, forming a positive feedback loop, is essential to promote tumor proliferation (Fig. 2).

In summary, m6A methylation promotes the formation of a hypoxic microenvironment, triggering a cascade of downstream biological reactions that influence immune cell functions and tumor biological behaviors. This intricate interplay significantly impacts the onset and progression of malignancies [21, 56, 149, 150]. In bladder cancer, HIF-1α promotes the upregulation of m5C expression by activating ALYREF. This induction of glycolysis accelerates tumor growth, contributing to the establishment of a hypoxic tumor immune microenvironment (TIME) that facilitates immune evasion [97]. Addressing hypoxia represents an effective strategy to enhance the antitumor immune response [151].

#### Metabolic reprogramming

**Fig. 3:**
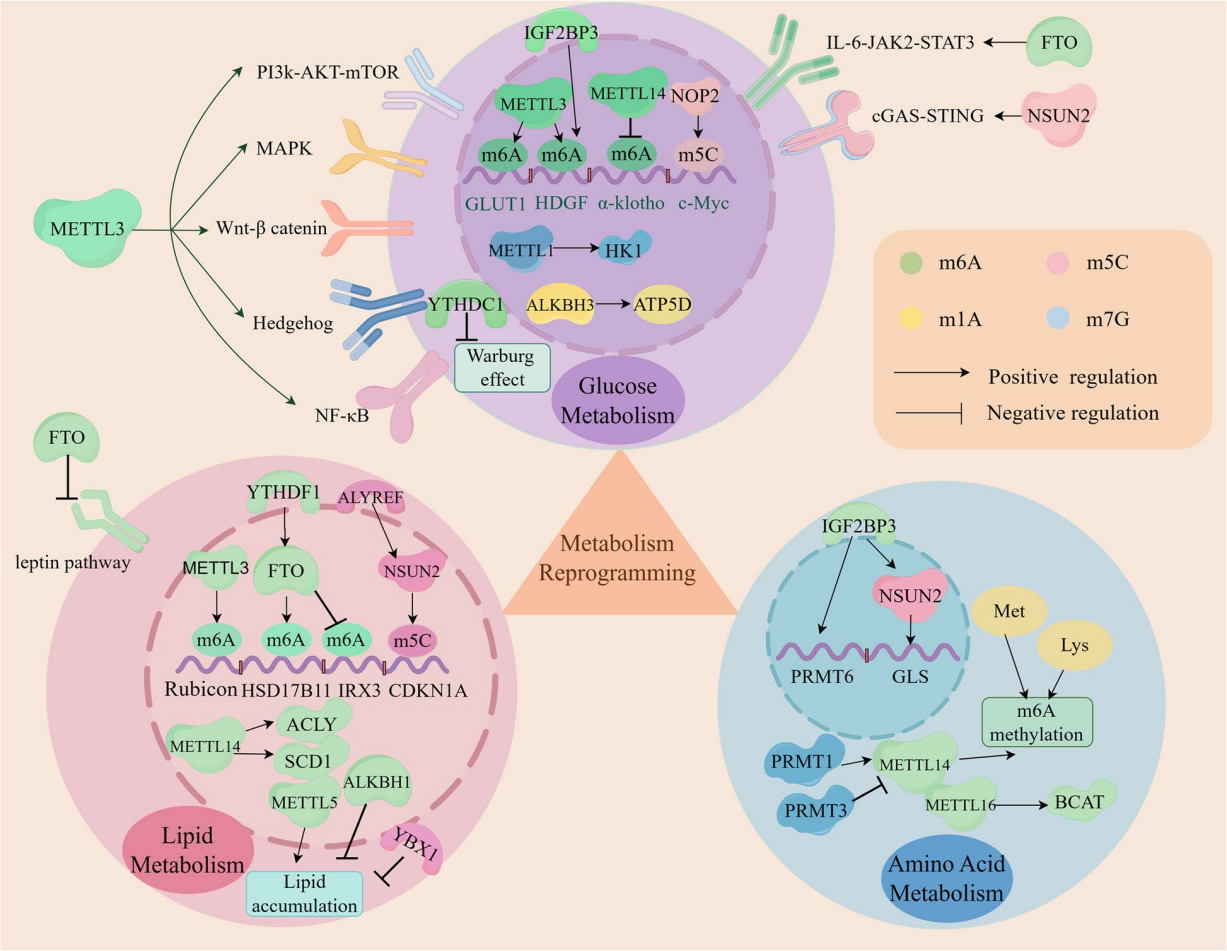
RNA methylations participate in metabolic reprogramming of the TME, including glucose metabolism, lipid metabolism and amino acid metabolism. RNA methylations regulate the expression of glycolysis-associated genes (GLUT1, Gys2, HDGF) and signal pathways (PI3K-AKT, mTORC1, MAPK, Wnt-β catenin, Hedgehog, NF-κB, IL-6/JAK2/STAT3, cGAS/STING) and enhance Warburg effect through their regulators, such as METTL1, METTL3, METTL14, NOP2, NSUN2, FTO, ALKBH3, IGF2BP3, YTHDC1 and. m6A and m5C accelerate lipid accumulation. m6A, m5C and m7G modulate the metabolisms of glutamine, arginine, methionine and lysine. These methylations impact tumor cell immunogenicity, proliferation, immune escape as well as tumor progression. ACLY, ATP citrate lyase; SCD1, stearoyl-CoA desaturase1; BCAT1, branched-chain amino acid transaminase 1; Met, methionine; Lys, lysine; PRMT1, protein arginine methyltransferase1. Figure created with figdraw.com

Metabolic reprogramming stands out as a significant mechanism for tumor immune evasion [151]. The process of RNA modification within metabolic reprogramming encompasses three types of metabolites: glucose, lipid, and amino acids (Fig. 3). Extensive evidence has illustrated that RNA methylation regulates the homeostasis of TME through these three substance metabolisms, subsequently influencing tumor immune evasion [135] (Fig. 2).

##### Glucose metabolism

Glucose metabolism serves as a pivotal pathway for tumor cells. A notable metabolic trait, termed the Warburg effect, describes the preference of tumor cells for glycolytic pathways over oxidative phosphorylation (OXPHOS), even in oxygen-rich environments [152]. This metabolic signature is closely intertwined with the immune functions of the TIME, impacting the biological characteristics of various immune cells, including activated T cells, dendritic cells (DCs), natural killer (NK) cells, and M1 macrophages. Furthermore, cancer cells can outcompete immune cells for nutrients, thereby suppressing the tumor immune response [153, 154].

Studies have demonstrated that m6A regulators promote glycolytic reprogramming through various glycolytic-associated genes and signaling pathways in multiple cancers [142]. For instance, METTL3 can induce GLUT1 mRNA translation and facilitate glucose uptake and lactate generation, thus activating mTORC1 signaling in colorectal cancer [46] (Fig. 3). Furthermore, METTL3 exerts a significant influence on the progression of colorectal cancer through glycose metabolism via an m6A-IGF2BP3-dependent mechanism [155]. Additionally, in gastric cancer, IGF2BP3 directly recognizes the m6A site on HDGF (Heparin Binding Growth Factor) mRNA, a process initiated by METTL3. This recognition promotes tumor angiogenesis and glycolysis [48] (Fig. 3). Additionally, METTL3 can also activates others signal pathways, including the mitogen activated protein kinase (MAPK) signaling pathway, the Wnt-β catenin pathway, the Hedgehog signaling pathway, the NF-κB signaling pathway, as well as METTL3-IGF2BP2-Gys2 (the liver-specific glycogen synthase) axis [156–161]. Consequently, glycolysis process accelerates, and hepatic glycogenesis continues, providing essential conditions for tumor proliferation (Fig. 3). There is evidence indicating that METTL14 efficiently utilizes glucose to induce glomerular endothelial cell injury by modifying m6A methylation, resulting in the downregulation of α-klotho expression [49, 162].

The demethylase FTO has been shown to be responsible for decreasing m6A methylation of Apolipoprotein E (APOE) mRNA and modulating the IL-6/JAK2/STAT3 signaling pathway, thereby inhibiting tumor glycolysis and abrogating tumor growth [163] (Fig. 3). Furthermore, the m6A reader YTHDC1 contributes to suppressing glycolysis by attenuating the Warburg effect, ultimately impeding pancreatic tumorigenesis [68].

It has been reported that NSUN2, the methylase responsible for m5C modification, can bind with glucose to sustain the oncogenic activity of tumor cells. This process occurs through the promotion of three prime repair exonuclease 2 (TREX2) mRNA expression and activation of the cGAS/STING pathway, thereby mediating immunotherapy resistance [164]. Additionally, NOP2 can enhance glycolysis by upregulating the expression of glycolytic genes and increasing the m5C content of c-Myc mRNA [165].

Additionally, studies have demonstrated that ALKBH3, an m1A demethylase, positively regulates the translation of ATP5D mRNA, thereby accelerating glycolysis [67]. METTL1 has also been found to upregulate the expression of the glycolysis rate-limiting enzyme HK1 [69]. Numerous pieces of evidence highlight the critical role of RNA methylation regulators in cancer cell glycolysis (Fig. 3).

##### Lipid metabolism

Fatty acids, as a significant metabolic pattern, play crucial roles in maintaining essential cellular physiological functions and participating in various cellular activities. Aberrant lipid metabolism has emerged as a key factor in tumorigenesis [166]. Dysregulated lipid metabolism not only suppresses the anti-tumor capabilities of immune cells but also facilitates immune evasion by cancer cells, thus impairing the immune response and reshaping the immunosuppressive TME. This alteration is characterized by both catabolic and anabolic processes closely associated with tumor immune evasion [167, 168]. Lipid metabolism encompasses processes such as synthesis, degradation, and storage of lipids. Tumor cells utilize these metabolites for membrane assembly and energy generation, significantly contributing to tumor cell proliferation [168].

Several pieces of evidence suggest that RNA methylation plays a crucial role in lipid metabolism in various cancers. Specifically, research indicates that YTHDF1 can bind to m6A-marked Rubicon mRNA, a process mediated by METTL3, ultimately impeding the fusion of autophagosomes with lysosomes and obstructing the clearance of lipid droplets (LDs) [169]. Additionally, overexpression of METTL14 enhances the protein levels of ATP citrate lyase (ACLY) and stearoyl-CoA desaturase 1 (SCD1), leading to increased production of triglycerides and cholesterol and accumulation of LDs [170] (Fig. 3). Moreover, the demethylase FTO promotes the formation of LDs in EC cells by facilitating the expression of the HSD17B11 gene via a YTHDF1-dependent mechanism [171]. Additionally, FTO enhances adipogenesis and fat deposition while inhibiting lipolysis by suppressing IRX3 expression and the leptin pathway, thereby promoting the progression of lipid disorder diseases [172] (Fig. 3). However, the demethylase ALKBH1 reduces the uptake and synthesis of lipids, leading to a decrease in hepatic lipid accumulation, thereby alleviating hepatic steatosis and the progression of nonalcoholic fatty liver disease (NAFLD) [173]. In vitro and mouse models have shown that METTL5 knockdown significantly reduces the levels of triglycerides, cholesterol, and intracellular free fatty acids, effectively blocking the progression of HCC [174]. Knockdown of NSUN2 decreases the protein expression of cyclin-dependent kinase inhibitor 1A (CDKN1A) in a m5C-ALYREF-dependent manner, indicating that the NSUN2-m5C-ALYREF signaling pathway plays a significant role in suppressing adipogenesis [81]. Similarly, m5C inhibits adipogenesis via the ALYREF-m5C-YBX2 and ALYREF-m5C-SMO pathways [175]. These findings suggest that various RNA modification proteins regulate the lipid metabolism of cancer cells through multiple mechanisms and signaling pathways, potentially serving as promising therapeutic targets and providing a research direction for immunotherapy.

##### Amino acid metabolism

Abnormal amino acid metabolism has been shown to suppress the anti-tumor immune capacity of immune cells and mediate tumor immune evasion [176]. Specifically, the reprogramming of glutamine metabolism plays a vital role in the anti-tumor immune response within TME [177]. Glutamine synthesis, as a critical proliferative metabolite, is widely upregulated in cancer-associated fibroblasts (CAFs) and is essential for lymphocyte proliferation, protein synthesis, and antibody production. Studies have demonstrated that blockade of glutamine metabolism alleviates the immunosuppressive TME and overcomes tumor immune evasion, ultimately inhibiting tumor growth [178, 179].

In the context of AML, branched-chain amino acid (BCAA) transaminase 1 (BCAT1) and BCAT2 drive carcinogenesis by reprogramming BCAA metabolism. METTL16 promotes BCAT expression in an m6A-dependent manner, thereby regulating metabolism to facilitate cancer progression [180]. Additionally, IGF2BP2 recognizes m6A to regulate the expression of critical targets in glutamine metabolism, making it a potential therapeutic target in AML [181]. Moreover, IGF2BP3 stabilizes PRMT6 (protein arginine methyltransferase 6) mRNA, which in turn mediates histone H3R2me2a methylation and maintains the function of leukemia stem cells (LSCs) [182, 183]. Additionally, PRMT3 interacts with METTL14 and is involved in its arginine methylation, leading to the downregulation of METTL14 expression levels. Depletion of PRMT3 enhances sensitivity of EC cells to ferroptosis by increasing m6A levels of Glutathione peroxidase 4 (GPX4) mRNA [184]. METTL14 also recognizes histone H3 trimethylation at lysine-36 (H3K36me3) to interact with the m6A methyltransferase complex (MTC) and affect m6A methylation [185]. Furthermore, Protein arginine N-methyltransferase 1 (PRMT1) catalyzes the methylation of METTL14 at arginine 255 (R255), stabilizing the m6A methyltransferase complex METTL3/METTL14 and facilitating m6A methylation [186].

It has been shown that metabolites originating from methionine metabolism contribute to m6A methylation and the translation of immune checkpoints. Furthermore, restricting methionine in the diet inhibits tumor growth and improves the anti-tumor immune response by enhancing the abundance and cytotoxicity of CD8^+^ T cells [187] (Fig. 3).

Therapies utilizing glutamine blockade to inhibit tumor cell metabolism have been proposed; however, these approaches equally damage immune cell metabolism, and as of yet, none have been approved for practical application [188]. Furthermore, depletion of the m6A-specific reader YTHDF1 in combination with PD-1 blockade has shown enhanced efficacy in anti-tumor therapy. A low protein diet supplemented with methionine and lysine has been found to enhance the expression of m6A and reduce the expression of FTO and ALKBH5, possibly through regulation by the transcription factor PPARγ [189]. Additionally, NSUN2-methylated lncRNA enhances the stability of glutaminase (GLS) mRNA by upregulating glutaminase expression through interaction with the IGF2BP3/HUR complex, thus facilitating reprogramming of glutamine metabolism and accelerating gastric cancer progression [190] (Fig. 3). In m7G-associated molecular subtypes of sepsis, subtypes with higher amino acid metabolism activity are characterized by more abundant activated macrophages, M0 and NK cells, and higher expression of immune regulatory genes [191]. Not only is RNA methylation able to regulate multiple types of amino acid metabolism, but conversely, amino acid metabolism plays a critical role in RNA methylation [70].

Taken together, abnormal metabolism can result in immune system dysfunction, tumor oncogenesis, progression, invasion, and immune evasion. The hypoxic microenvironment promotes glycolysis, exacerbating tissue hypoxia. Methylation, hypoxia, and glycolysis form a positive feedback loop that impacts various downstream responses (Fig. 2). These aberrant conditions suppress immune cell functions and promote tumor biological behavior.

### RNA methylation regulates tumor innate immunity

The oncogenic process triggers the host innate immunity, which encompasses a variety of immune cells, including macrophages, monocytes, neutrophils, myeloid-derived suppressor cells (MDSCs), dendritic cells (DCs), and others. The characteristics of these immune cells are also influenced by features of the TME, such as hypoxia and metabolic abnormalities [192, 193]. Therefore, we will explore several immune cells closely associated with RNA methylation and tumor innate immunity.

### Tumor-associated macrophages

Macrophages play a critical role in the immune response, encompassing both innate and adaptive immunity through activities such as phagocytosis of foreign material, antigen presentation, and secretion of proteins and cytokines across various phenotypes [194]. Tumor-associated macrophages (TAMs) represent a major infiltrating cell type within tumors and contribute significantly to the formation of the tumor microenvironment [195, 196]. TAMs originate from bone marrow monocytes, including resident macrophages and circulating monocytes recruited to the TME [197]. M-MDSCs (monocyte-related myeloid-derived suppressor cells) serve as the primary circulating precursors of TAMs and can be induced into TAMs by chemokines, as well as by the immunosuppressive programming of MDSCs [198].

TAMs are typically categorized into two distinct functional subtypes: classical activated M1 macrophages and alternatively activated M2 macrophages [199]. These infiltrating macrophages are widely considered to be involved in various aspects of tumorigenesis, including progression, invasion, angiogenesis, metastasis, and drug resistance [199, 200]. High levels of infiltration are closely associated with poor prognosis and therapeutic response, including targeted therapy, radiotherapy, and chemotherapy [201]. Within the TME, elements such as fibrosis, hypoxia, metabolic reprogramming, and cytokines contribute to the phenotypic variation of TAMs, inducing polarization toward M1/M2 phenotypes [195]. Initially, macrophages exhibit a pro-inflammatory M1 secretion profile during the early healing stage, which transitions to an anti-inflammatory M2 secretory profile in the later stage [195]. While M1 macrophages are generally considered anti-tumorigenic, and M2 macrophages are considered pro-tumorigenic [195, 202]. It's worth noting that M1 macrophages can also express M2 markers and vice versa [203]. TAMs demonstrate a high degree of plasticity, capable of polarizing pro-tumor M2-type macrophages into M1 TAMs and altering their functions, thereby exerting a role in suppressing tumor progression [204].

**Fig. 4:**
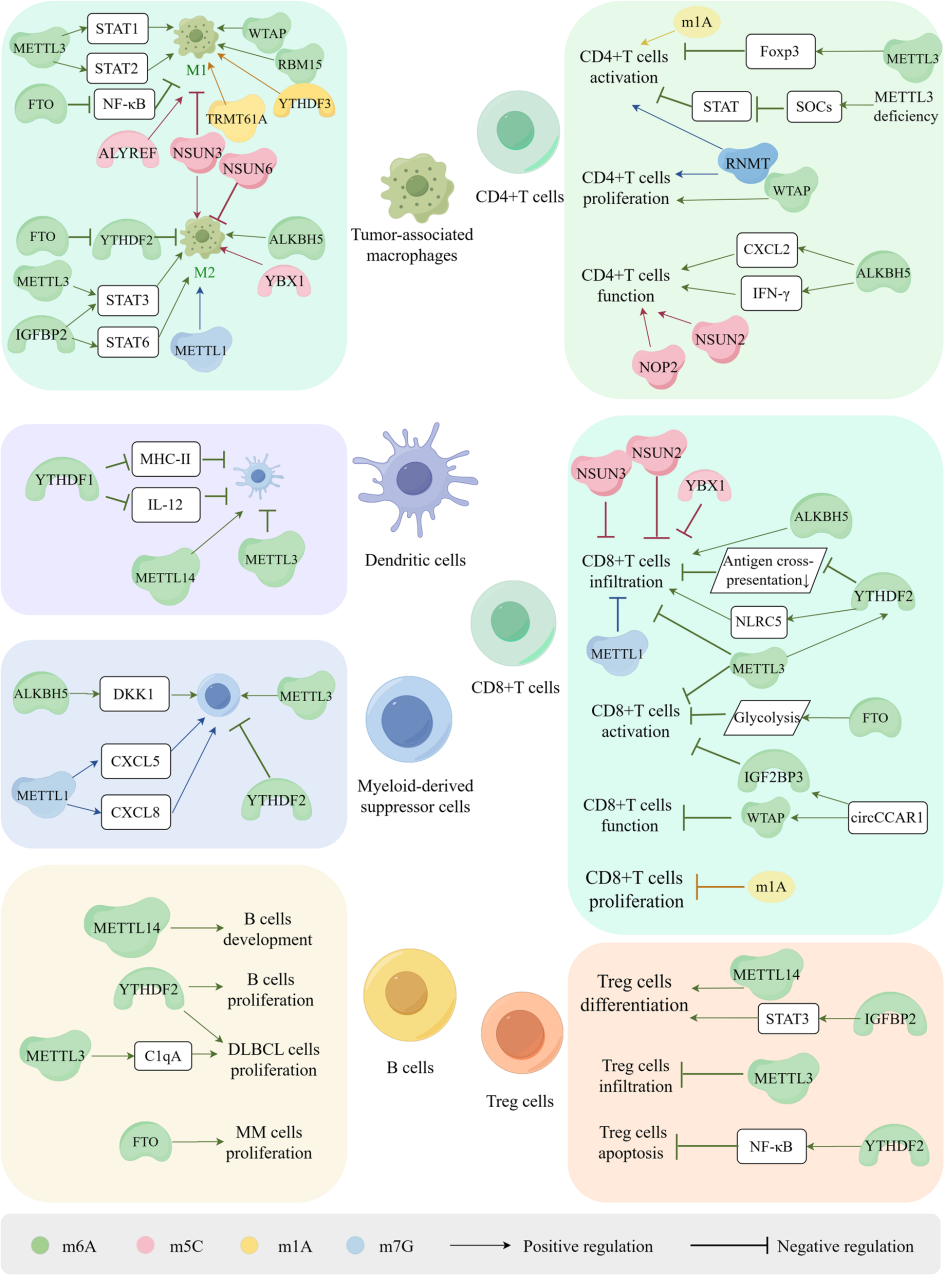
Mechanisms of RNA methylations regulate of the biological functions of immune cells in the TME, including immune cell differentiation, development, infiltration, activation, proliferation and apoptosis. RNA methylations promote tumor-associated macrophages (TAMs) polarization towards M1 macrophage or M2 macrophage and regulate the proliferation and infiltration of dendritic cells (DCs), Myeloid-derived suppressor cells (MDSCs) and regulatory T (Treg) cells. Furthermore, RNA methylations play a significant role in the differentiation and development of T cells. m6A and m5C suppress the infiltration and activation of CD8^+^ T cells as well as mediating their dysfunction. m1A and m7G also participate in the activation, infiltration and proliferation of CD4^+^T cells and CD8^+^T cells, however, the regulators of m1A and m7G in these processes remain further investigation. RNA methylations regulate tumor immune response and evasion through impacting various biological functions of immune cells, such as the differentiation, development, infiltration, activation, proliferation and apoptosis of immune cells. Figure created with figdraw.com

Research has demonstrated that RNA methylation regulates macrophage polarization through reprogramming of the TME and various signaling pathways [204]. METTL3 plays a crucial role in macrophage polarization [22]. Yin et al. showed that depletion of METTL3 increased the expression of M1/M2-associated genes and promoted the polarization of bone marrow-derived macrophages (BMDMs) toward both M1 and M2 TAMs via NF-κB and STAT3 pathways, thereby enhancing the infiltration of TAMs into tumors [205, 206]. In models with METTL3 depletion, the therapeutic efficacy of PD-1 blockade was reduced, leading to accelerated tumor progression and distant metastasis [205]. Shu et al. demonstrated that METTL3 drove M1 polarization of macrophages and accelerated liver fibrosis through m6A methylation [207]. Similarly, Liu et al. found that upregulation of METTL3 expression was accompanied by an increase in M1 macrophages and a decrease in M2 macrophages, a process mediated by STAT1 mRNA [208]. Furthermore, lactic acid facilitated M2 polarization by activating METTL3 via the Trib1/ERK/STAT3 pathway [209]. Knockdown of METTL3/METTL14 significantly inhibited macrophage activation and secretion and slowed the progression of liver fibrosis [210, 211]. Additionally, WTAP and RBM15 interact with M1 macrophages and mediate downstream inflammatory responses [212] (Fig. 4).

Knockdown of the demethylase FTO inhibited the polarization of both M1 and M2 macrophages by dysregulating the expression of STAT1 in M1 macrophages and STAT6 in M2 macrophages. This dysregulation occurred via suppression of the NF-κB signaling pathway and silencing of YTHDF2 [212]. Additionally, knockdown of ALKBH5 resulted in decreased infiltration of M2 macrophages [59, 213]. Studies have indicated that IGFBP2 plays a crucial role in shifting M1 macrophages towards M2 polarization through the STAT3 or STAT6 pathways, thereby contributing to the formation of an immunosuppressive microenvironment [196, 214, 215] (Fig. 4).

The polarization of TAMs is also regulated by other RNA modifications. In a prognostic score model, NSUN3 knockdown has been shown to decrease the infiltration of M2 macrophages while increasing the infiltration of M1 macrophages [216, 217]. Intriguingly, NSUN6 inhibits the expression of macrophage-associated chemokines by promoting HDAC10 expression, thereby suppressing the recruitment of M2 macrophages and improving prognosis in bladder cancer patients [218]. High expression of YBX1 is associated with the infiltration of M2 macrophages and T cell depletion, which could potentially be targeted using M1 polarization agents in synergy with immunotherapy [219]. In Abdominal Aortic Aneurysm (AAA), immune infiltration analysis has shown that YTHDF1/2/3, YTHDC1, RRP8, and TRMT61A are upregulated genes associated with the infiltration of M1 macrophages, while FTO and ALKBH1 are downregulated [220].

The m1A reader, YTHDF3, facilitates the polarization of M1 macrophages and exacerbates inflammation [220]. ALKBH3-mediated m1A demethylation stabilizes the cytokine macrophage colony-stimulating factor (CSF-1) mRNA, promoting the progression of breast and ovarian cancer [66].

Moreover, m7G methylation is positively correlated with the abundance of M2 macrophages [69]. METTL1 also plays a role in the polarization of TAMs. Elevated METTL1 expression correlates with increased infiltration of M2-like macrophages, while inhibition of METTL1 and decreased m7G methylation of tRNAs induce TAMs towards an M1-like endotype in preclinical models of PCa [128]. Data from The Cancer Genome Atlas (TCGA) database indicates that ALYREF, ZC3H13, WTAP, and METTL1 are negatively associated with M1 macrophages [221] (Fig. 4).

Taken together, these findings underscore the significant role of RNA methylation in the polarization of TAMs. These RNA methylation regulators have the ability to catalyze and modulate the phenotypes of TAM polarization, thereby influencing the infiltration of TAMs within tumors and ultimately shaping the immunosuppressive microenvironment. Moreover, these insights provide novel targets and strategies for immunotherapy.

### Dendritic cells

Dendritic cells (DCs) are pivotal antigen-presenting cells that play a crucial role in both innate and adaptive immune responses [222]. As part of the antigen-presenting cell (APC) population, which also includes macrophages and B lymphocytes, DCs are capable of uptaking, processing, and presenting antigens to T cells [223]. However, within the TME, the function and activity of DCs are regulated by immunosuppressive factors and interactions with other immune cells, potentially leading to immune evasion and exacerbating oncogenesis [223, 224].

Recent studies have shed light on the involvement of m6A methylation in DC-mediated anti-tumor responses. Knockdown of YTHDF1 has been shown to enhance the expression of MHC-II on DCs and increase the secretion of interleukin-12 (IL-12), thereby bolstering adaptive immune responses [225]. METTL3 has been implicated in the regulation of DC activation and the mediation of immune dysfunction through m6A methylation [23, 226] (Fig. 4). Additionally, the tumor suppressor gene METTL14 is positively correlated with DCs, and its knockdown has been found to promote immunosuppression in breast cancer [227, 228]. Researchers have also demonstrated that the m6A-YTHDF1 axis restricts the cross-priming capacity of DCs, and loss of YTHDF1 enhances antigen presentation capacity [229]. The infiltration of DCs has been correlated with the ALKB family; however, further exploration is warranted to elucidate the interaction between them [230].

### Myeloid-derived suppressor cells

As a significant component of TME, myeloid-derived suppressor cells (MDSCs) originate from the bone marrow and serve as precursors to dendritic cells, macrophages, and granulocytes. These cells possess the ability to inhibit T cell-mediated immune responses, thereby impacting cancer outcomes [231, 232]. Studies have revealed that expression levels of METTL3 are closely associated with the expansion of MDSCs, and loss of METTL3 inhibits the accumulation and immunosuppressive capacity of MDSCs, resulting in increased infiltration of CD4^+^ and CD8^+^ T cells [233, 234]. Furthermore, the expansion and suppressive function of MDSCs are enhanced in YTHDF2-knockout mice [235, 236]. Additionally, ALKBH5 facilitates MDSCs accumulation by inducing the expression of Dickkopf-related protein 1 (DKK1) [58, 237] (Fig. 4). Moreover, METTL1 upregulates the expression of chemokines CXCL5 and CXCL8 in an m7A-dependent manner, leading to MDSCs accumulation and immunosuppression in HCC and intrahepatic cholangiocarcinoma (ICC) [238, 239] (Fig. 4).

### RNA methylation regulates tumor adaptive immunity

RNA methylation has emerged as a critical regulator of adaptive immunity, shaping the outcome of the host immune response [240, 241]. Adaptive immunity in tumor immune responses primarily involves T lymphocytes and B lymphocytes. Research indicates that RNA methylation plays a pivotal role in the development, differentiation, activation, exhaustion processes, and therapeutic responses of these immune cells by modulating the translation and expression of RNA and proteins [242]. Below, we delve into the specific regulatory mechanisms of RNA methylation in adaptive immunity and immune cells.

### T lymphocytes

T lymphocytes, critical components of adaptive immunity, originate from bone marrow progenitors and undergo maturation in the thymus, where they play pivotal roles. Naïve T cells possess the ability to differentiate into various subsets, such as T helper (Th) cells, depending on their stem cell features [243]. During thymic development, T cell precursors undergo positive or negative selection, leading to differentiation into CD4^+^ or CD8^+^ T cells in the thymic cortex and regulatory T (Treg) cells in the thymic medulla [244]. Numerous studies have highlighted the role of RNA methylation in mediating various functions of T cells, including proliferation, activation, and apoptosis, through the involvement of multiple RNA methylation regulators [245, 246] (Fig. 4).

#### *CD4*^+^*T cells*

Researchers have demonstrated that inhibiting METTL3 facilitates the activation of CD4^+^ T cells while suppressing the differentiation of effector T cells, particularly Treg cells, by reducing the expression of Foxp3 in a m6A-dependent manner [247]. Inhibition of METTL3 reduces m6A methylation levels, promotes cell apoptosis, hinders effector T cell differentiation, and inhibits allogeneic CD4^+^ T cell responses [24]. In naïve T cells deficient in METTL3, the activity of the SOCs family is enhanced, which encodes STAT inhibitory proteins, thus suppressing STAT activation and impeding the proliferation and differentiation of T cells [248]. Similarly, WTAP and METTL3 exhibit similar characteristics in regulating mRNA stability. CD4^+^ T cells deficient in WTAP undergo apoptosis and exhibit reduced proliferation upon TCR signal activation [249]. The presence of m6A methylase is essential for T cells to exert immune functions. Additionally, the m6A demethylase ALKBH5 enhances the stability of CXCL2 and IFN-γ mRNA and proteins by reducing m6A modification expression, thereby preserving CD4^+^ T cell immune function [250] (Fig. 4).

During HIV-1 infection of CD4^+^ T cells, m6A levels are upregulated, potentially mediated by variations in the activity of m6A writers or erasers in T-cells [251, 252]. Overexpression of YTHDF3 has been shown to decrease the production and infection of HIV-1 by incorporating into viral particles [253, 254]. Evidence suggests that NOP2 promotes m5C methylation in HIV-1 and interacts with TAR by competing with Tat protein, thereby inhibiting HIV-1 replication and transcription, prolonging the incubation period [255]. Additionally, IL-17 treatment reduces the posttranslational modification of YBX1 in CD4^+^ T cells, inhibiting HIV infection by suppressing HIV reverse transcription [256].

In patients with Systemic lupus erythematosus (SLE), the levels of m5C and NSUN2 expression are decreased in CD4^+^ T cells, and hypermethylated m5C is involved in immune-related and inflammatory pathways, including the immune system, cytokine signaling, and interferon (IFN) signaling [257]. m7G methylation is essential for T cell activation. RNMT, a key regulator of T cell activation, controls ribosome generation, enhances mRNA translation efficiency, and promotes proliferation and differentiation [258]. Although tRNA modification is a dynamic process during T cell activation, the m1A methylation at position 58 of tRNA remains constant, suggesting its involvement in the translation of T cell activation [259] (Fig. 4).

#### *CD8*^+^*T cells*

Numerous studies have highlighted a close association between RNA methylation and the infiltration of CD8^+^ T cells in cancers [260–262] (Fig. 4). Tumors exhibiting high m6A expression demonstrate stronger immunogenicity by increasing HLA-A content, which enhances immunosurveillance and activates immune cell infiltration [263]. For instance, YTHDF2 depletion enhances the activation and antitumor response of CD8^+^ T cells by augmenting their antigen cross-presentation ability and the abundance of infiltrating immune cells [229, 264, 265]. Moreover, METTL3 knockdown inhibits the generation of MDSCs, leading to the activation and proliferation of CD4^+^ and CD8^+^ T cells [234]. Conversely, a study has shown that METTL3 overexpression increases CD8^+^ T cell proportions, attenuates immune evasion, and inhibits the progression of EC by promoting m6A modifications of NLRC5 via a YTHDF2-dependent mechanism [47]. Evidence has shown that IGF2BP3 inhibits the activation of CD8^+^ T cells and facilitates tumor immune evasion [61, 266]. A recent study has demonstrated that exosome-derived circCCAR1 upregulates WTAP expression by binding with IGF2BP3, thereby enhancing its stability through increased m6A expression. CircCCAR1 can be ingested by CD8^+^ T cells, causing them to malfunction by stabilizing the PD-1 protein [267]. Furthermore, tumor cells utilize glycolysis promoted by FTO to inhibit the activation and effector states of CD8^+^ T cells, which can be reversed by combining an FTO inhibitor with anti-PD-L1 blockade [268]. These findings suggest a promising therapeutic strategy for multiple types of cancers. However, as an m6A demethylase, elevated levels of ALKBH5 have been shown to enhance the infiltration of CD8^+^ T cells [269]. The mechanisms underlying the relationship between demethylases and the activation of CD8^+^ T cells require further exploration.

NSUN2 boosts m5C methylation to stabilize TREX2 mRNA, reducing the infiltration of CD8^+^ T cells and fostering resistance to anti-PD-L1 immunotherapy through activation of the cGAS/STING pathway [164]. Additionally, NSUN3 expression inversely correlates with the infiltration of CD8^+^ T cells [217, 270]. Knockdown of the m5C reader YBX1 decreases the infiltration of MDSCs and Tregs while increasing the infiltration of CD8^+^ T cells, thereby enhancing the anti-tumor immune response [271]. m1A negatively regulates the proliferation of CD8^+^ T effector cells in colon cancer [272]. Similarly, high expression of m7G is associated with decreased cytotoxic CD8^+^ T cell infiltration and increased M2 macrophage infiltration [69, 128, 273] (Fig. 4). Together, these findings suggest that RNA methylation could be a promising therapeutic target for enhancing the tumor immune response.

#### Treg cells

m6A methylation has been demonstrated to regulate the proliferation of immunosuppressive Treg cells [43]. METTL14 deficiency inhibits the differentiation of naïve T cells into Treg cells, and METTL14-deficient Treg cells exhibit impaired function in suppressing inflammation induced by naïve T cells. However, adoptive transfer of Treg cells can alleviate this impaired function [274, 275]. Additionally, there is a negative correlation between METTL3 expression levels and Treg infiltration [276]. Insulin-like growth factor binding protein 2 (IGFBP2) contributes to the activation of the STAT3 signaling pathway, leading to Treg differentiation and the creation of a suppressive tumor environment [277]. Studies have shown that the loss of YTHDF2 in Tregs promotes Treg apoptosis and suppresses their function in the TME, thereby inhibiting tumor progression through the YTHDF2-m6A-NF-κB pathway [278, 279] (Fig. 4).

### B lymphocytes

B lymphocytes are integral to the adaptive immune response, functioning by producing antibodies, which include memory B cells and plasma cells [280]. Evidence has verified that RNA methylation and its regulatory factors are involved in various B cell-associated diseases [281, 282]. RNA m6A methylation plays a critical role in the development, maturation, and antibody secretion of B cells [281, 283–285] (Fig. 4). The deletion of METTL14 constrains the development from large pre-B cells to small pre-B cells by reducing m6A methylation levels, and the deletion of YTHDF2 results in a significant block of pro-B cell proliferation [283]. Studies have shown that METTL3 inhibits the complement pathway by mediating C1qA methylation and enhances resistance to Rituximab, thereby facilitating the progression of diffuse large B-cell lymphoma (DLBCL) [286]. In AML, METTL3 also plays a role in pre-B cell to macrophage trans-differentiation, and this effect can be inhibited by the METTL3 inhibitor [287]. The writer KIAA1429 also plays a role in DLBCL progression [55]. Additionally, YTHDF2 can identify m6A sites on alkaline ceramidase 2 (ACER2) mRNA, promoting the proliferation of DLBCL cells and contributing to disease progression [282]. METTL14-mediated YTHDF2 activity facilitates the formation of germinal centers and regulates positive selection and cell cycle regulation of germinal center B cells in an m6A-dependent manner [288, 289]. Furthermore, the m6A reader YTHDF1 recognizes and destabilizes Epstein–Barr virus (EBV) mRNA, thereby suppressing EBV infection and replication, which is significant in B-cell malignancies [290]. Expression levels of m6A are decreased in plasma cells of patients with multiple myeloma (MM) due to FTO-mediated demethylation, and inhibiting FTO suppresses MM cell proliferation, migration, and invasion [291].

Accordingly, RNA methylation serves a crucial role in both innate and adaptive immune responses, influencing various biological processes within immune cells. These include guiding macrophage polarization towards the M2 phenotype, promoting the accumulation of MDSCs, affecting the function of DCs in antigen presentation, reducing the infiltration and activation of effector T cells, influencing the differentiation of Tregs, and contributing to abnormal proliferation of B cells.

### RNA Methylation Mediates Tumor Immune Evasion

The tumor microenvironment is distinguished by an immunosuppressive state that is instrumental in both the downregulation of immune cell functions and the facilitation of tumor immune evasion [135, 292]. This evasion significantly contributes to the creation of an immunosuppressive environment that not only promotes oncogenesis but also allows for its uncontrolled proliferation [293]. Antitumor responses primarily involve activated CD8^+^ T cells, which specifically recognize and target tumor antigens presented by APCs. These cells then exert cytotoxic effects to destroy tumor cells [294]. However, tumor cells have the ability to emit suppressive signals that impair the immune functions of T cells, thus hindering effective immune responses [293].

The immune system is critical in mounting anti-tumor responses. Yet, tumor cells often evade immune surveillance and elimination via various mechanisms, such as creating an immunosuppressive TME, downregulating HLA-1, and upregulating immune checkpoint proteins [295, 296]. Tumor immune evasion is characterized by the continuous and uncontrolled expansion of the tumor immune microenvironment [293]. Tumor cells manipulate intrinsic regulators to forge an immunosuppressive microenvironment and alter tumor metabolism, thereby impairing immune cell functions and promoting immune evasion [297, 298]. Furthermore, the interaction between PD-1 and PD-L1 facilitates tumor evasion of immunosurveillance by fostering immune tolerance and curtailing the proliferation, survival, and effector functions of CD8^+^ cytotoxic T lymphocytes (CTLs), as well as triggering apoptosis in tumor-infiltrating T cells [299]. The aforementioned details highlight the role of RNA methylation in enhancing hypoxic and metabolic reprogramming within tumors.

**Fig. 5:**
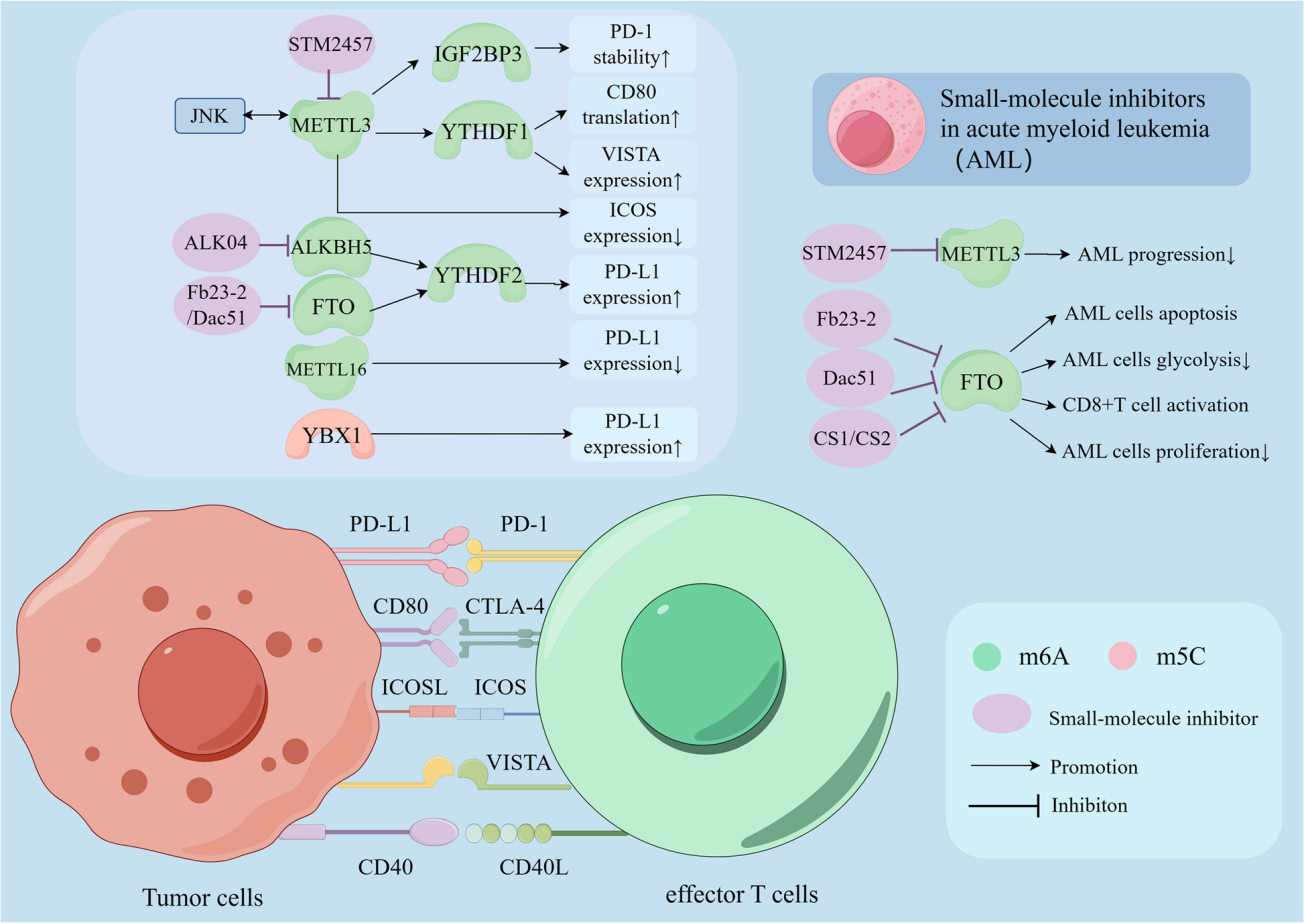
RNA methylations regulate expression of immune checkpoints through their regulators, and several small-molecule inhibitors combined with immune checkpoint blockade are applied in acute myeloid leukemia (AML). Co-inhibitory receptor-ligand complexes includes PD-1/PD-L1, CTLA-4/CD80, VISTA and so on. Co-stimulatory receptor-ligand complexes includes CD40/CD40L, ICOS/ICOSL and so on. m6A and m5C regulate the expression, translation, and stability of immune checkpoints as well as their sensibilities to immunotherapy. Immune checkpoints such as PD-1, CTLA-4, ICOS, VISTA, CD40L bind with their respective ligands on tumor cells, triggering a negative or positive signal to T cells response. This process can be impacted by several regulator proteins of RNA methylations, such as METTL3, ALKBH5, FTO and METTL16. Several small-molecular inhibitions targeting METTL3 and FTO, including STM2457, Alk-04, FB23-2, Dac51 and so on, can inhibit m6A methylation process and can be applied in AML. Figure created with figdraw.com

RNA methylation plays a pivotal role in regulating tumor immunosuppressive factors, thereby modulating tumor immune evasion mechanisms. For instance, m6A methylation significantly influences the regulation of PD-1/PD-L1 through mechanisms such as splicing, stability, and translation, ultimately facilitating immune evasion [300, 301]. Specifically, m6A methylation enhances PD-1/PD-L1 expression via the METTL3-JNK signaling pathway [302]. In this pathway, JNK interacts with and binds to METTL3, which increases the m6A modification of mRNA, thereby elevating PD-1 levels and reducing the cytotoxic effectiveness of CD8^+^ T cells, leading to tumor immune evasion [302]. Moreover, the expression of PD-L1 is linked to both METTL3 and IGF2BP3; the latter recognizes m6A sites and blocks PD-1 degradation to promote immune evasion [25, 303]. Additionally, METTL3 is known to augment the immunosuppressive abilities of tumor-infiltrating myeloid cells [304]. In the context of EC, Serine hydroxymethyltransferase 2 (SHMT2) utilizes the METTL3/FTO/ALKBH5/IGF2BP2 pathway to mediate immune evasion by modifying c-myc through m6A [305]. These findings further indicate that IGF2BP3 plays a crucial role in the regulation of PD-1/PD-L1 degradation and impacts tumor immune responses. Moreover, overexpression of METTL16, by decreasing mRNA stability via m6A modification, cooperatively inhibits tumor immune evasion along with PD-1 suppression [50]. Deficiencies in ALKBH5 or FTO can also suppress PD-L1 expression by hindering YTHDF2-mediated mRNA stability [306, 307]. Additionally, YTHDF1 promotes tumor immune evasion by enhancing PD-L1 expression [308] (Fig. 5). The expression of PD-L1 is upregulated by the m5C reader protein YBX1, which when interacting with PD-1, can significantly inhibit the proliferation and function of cytotoxic CD8^+^ T cells. This interaction thereby suppresses the immune response in patients [309]. These findings underscore the critical role of RNA methylation in facilitating tumor immune evasion, highlighting the potential of targeting this biochemical process as a promising therapeutic strategy.

### Targeting RNA Methylation Enhances the Therapeutic Effects of Immune Checkpoint Blockade

Immune checkpoint blockade (ICB) has shown significant success in clinical trials and has been approved for the treatment of various cancers. These include GC, HCC, CRC, NSCLC, SCLC, triple-negative breast cancer, urothelial carcinoma, melanoma, etc. [310–322]. Immune checkpoint inhibitors (ICIs) are designed to block the function of immune checkpoints, effectively alleviating the immunosuppressive state of T cells, reversing T cell exhaustion, and reactivating effector T cells within the TME. This action significantly boosts anti-tumor immune responses [323, 324]. Specifically, targeting PD-1 and its ligands, along with CTLA-4 the two principal immune checkpoints—has substantially improved outcomes in cancer treatment [323, 325–327]. Additionally, there is a growing body of evidence supporting the use of PD-L1 small-molecule inhibitors in combination with RNA modification modulators to enhance the effectiveness of ICB in clinical treatments [328, 329] (Table 2). Furthermore, the inhibition of methylases has been shown to significantly enhance the effectiveness of ICB therapy. For instance, inhibiting METTL1 has been demonstrated to improve responses to ICB therapy in preclinical models of PCa, and low expression of METTL1 is associated with favorable outcomes from ICB therapy [128].

**Table 2:** Small-molecule inhibitors targeting N6-Methyladenosine regulators and immune checkpoints

Small-molecule inhibitors	Target	Cancer type	References
STM2457	METTL3	CESC; AML	[330, 331]
FB23-2	FTO	AML	[332]
Dac51	FTO	AML	[332]
CS1	FTO	AML	[333]
CS2	FTO	AML	[333]
18097	FTO	Breast cancer	[334]
Alk-04	ALKBH5	CRC; Melanoma	[237]
Atezolizumab	PD-L1	SCLC; Triple-negative breast cancer	[311, 316]
Avelumab	PD-L1	Urothelial carcinoma	[320]
Durvalumab	PD-L1	SCLC	[321]
Nivolumab	PD-1	Advanced HCC	[310]
Ipilimumab	CTLA-4	Advanced Melanoma	[319]
Pembrolizumab	PD-1	Metastatic squamous cell carcinoma	[322]

*Abbreviation*: *AML* acute myeloid leukemia, *CESC* cervical squamous cell carcinoma, *CRC* colorectal cancer, *SCLC* small-cell lung cancer

The use of m6A regulator inhibitors in enhancing ICB therapies has been extensively explored in recent studies [335, 336]. m6A methylases play a crucial role in modulating the expression levels of PD-L1 and enhancing tumor sensitivity to anti-PD-1 and anti-CTLA-4 therapies, thereby improving the outcomes of ICB treatments [50, 234, 337]. Additionally, YTHDF1 is implicated in inducing resistance to ICIs by promoting the degradation of MHC-I molecules; inhibiting YTHDF1 can transform immunologically "cold" tumors into "hot" ones, making them more amenable to therapy [60]. YTHDF1 also contributes to the dysfunction of cytotoxic CD8^+^ T cells by encouraging the accumulation of MDSCs through IL-6 secretion, presenting a novel target for ICB immunotherapy [338]. Furthermore, both methionine metabolites and YTHDF1 are known to enhance the translation of immune checkpoints such as PD-L1 and VISTA, suggesting that targeting these processes could be an innovative strategy for ICB [187]. Depleting METTL3 in myeloid cells has been shown to reduce the efficacy of PD-1 blockade therapies by decreasing the translation efficiency of YTHDF1 [205]. Moreover, IGF2BP1 enhances PD-L1 mRNA stability and promotes tumor immune evasion by reducing CD8^+^ T cell-mediated cytotoxicity. This mechanism is potentiated by fibroblast growth factor receptor 4 (FGFR4), and targeting IGF2BP1 in conjunction with anti-PD-L1 therapy can inhibit the proliferation and invasion of HCC cells [339, 340].

Moreover, upregulation of m6A regulators has been observed in patients exhibiting resistance to immunotherapy. Notable among these regulators are METTL3, METTL16, ALKBH5, etc., suggesting their potential roles in the development of resistance mechanisms [341–343]. Overall, to enhance the efficacy of ICB in cancer immunotherapy, it is crucial to explore small-molecule inhibitors targeting RNA methylation regulators. This approach necessitates a thorough understanding of the complex interactions between immune checkpoints and RNA methylation mechanisms.

Several small-molecule inhibitors have been developed and are being used in conjunction with ICB (Fig. 5 and Table 2). Notably, STM2457, an inhibitor of METTL3, has been demonstrated to reduce m6A levels and inhibit the progression of AML [287, 330]. STM2457, when used in conjunction with anti-PD-1 antibodies, has been shown to significantly improve treatment outcomes in cervical squamous cell carcinoma (CESC) [331]. This METTL3 inhibitor is particularly noteworthy because it can eliminate AML cells without significantly harming normal hematopoiesis [330]. Additionally, substrate-competitive FTO inhibitors such as FB23-2 and Dac51 have been effective in promoting apoptosis in AML cells and reactivating CD8^+^T cells by inhibiting tumor glucose metabolism, respectively [332, 333]. Moreover, two other inhibitors, CS1 and CS2, have been documented to drastically reduce the proliferation of human AML cells by suppressing PD-L1 expression through the MYC pathway. Their therapeutic efficacy is reported to be over ten times greater than that of FB23-2 [334]. Another FTO inhibitor, named 18,097, has been successful in inhibiting the proliferation and migration of breast cancer cells and enhancing their chemosensitivity [344]. Furthermore, Alk-04, a specific inhibitor of ALKBH5, boosts the effectiveness of anti-PD-1 therapy and reduces the infiltration of Tregs and MDSCs in TME [237]. Beyond PD-1 and PD-L1, methylation regulators also affect other immune checkpoints such as CD80, ICOS, and VISTA. For instance, METTL3-mediated YTHDF1 recognition of m6A in CD80 transcripts enhances CD80 translation [23], and METTL3 deficiency correlates with reduced expression of the inducible co-stimulatory molecule (ICOS) [148]. YTHDF1 also increases the expression levels of PD-L1 and the PD-1 homolog VISTA [187]. Additionally, it has been reported that targeting modifications like m5C and m1A methylation can further enhance the effectiveness of ICB immunotherapy [345, 346]. These findings illustrate a broad and potent application of small-molecule inhibitors in cancer treatment, particularly when combined with established ICB strategies.

In conclusion, the inhibition of RNA methylation regulators is currently under investigation for its potential to curb tumor progression. Experimental evidence from animal studies has confirmed that combining immune checkpoint blockade with small-molecule inhibitors can effectively suppress tumor growth. The ongoing development and refinement of RNA methylation regulator inhibitors and ICIs are poised to yield significant advancements and offer promising new treatments for cancer patients in the foreseeable future.

## Conclusions and perspectives

In this review, we explored four types of RNA methylation and their regulatory roles: writers, erasers, and readers, within the TME. These regulators are involved in crucial biological processes including hypoxia and metabolic reprogramming, and they influence the development, differentiation, proliferation, infiltration, activation, and apoptosis of immune cells in tumor immunity. Furthermore, they mediate the expression of immune checkpoints, thereby facilitating tumor immune evasion. These modifications influence RNA fate through mechanisms such as splicing, transport, translation, stability, and degradation. Given these roles, RNA methylation significantly impacts the initiation, proliferation, invasion, and metastasis of cancer. By regulating the translation of immune checkpoints and mediating tumor immune evasion, these modifications highlight a promising area for targeting the interactions between RNA modification and immune checkpoints in cancer immunotherapy.

RNA methylation has been extensively studied for its varied biological functions, and its regulators have been widely examined in the context of cancer research. Interestingly, some regulators, such as METTL3, have been found to perform opposing functions depending on the disease type or even within different aspects of the same disease. For example, low expression of METTL3 is associated with resistance to anti-PD-1 antibodies in thyroid cancer [266], whereas inhibitors of METTL3 can improve treatment outcomes in AML [330]. These findings underscore the importance of thoroughly understanding the complex biological effects of methylation regulators in different cancers.

Overall, the prospects of RNA methylation in the field of cancer immunotherapy are promising. These regulators can be utilized to estimate the diagnosis and prognosis of cancer by assessing the upregulation or downregulation of expression levels. Furthermore, there is potential to exploit cancer vaccines targeting the regulators' functions in tumor immunity, as RNA methylation plays a crucial role in regulating RNA fate. These regulators also modulate the function of immune cells, the invasion capacity of tumor cells, and the expression of immune checkpoints, thereby influencing tumor progression, resistance, and recurrence. In conclusion, targeting these biological functions and developing more small-molecule inhibitors, especially in combination with ICB immunotherapy, holds great promise for clinical treatment and offers encouraging prospects in the field of cancer immunotherapy.

## Data Availability

Not applicable.

## Abbreviations

AAA: Abdominal Aortic Aneurysm
ACC: Adrenocortical carcinoma
ACER2: Alkaline ceramidase 2
ALKBH1: α-Ketoglutarate-dependent dioxygenase ABH1
ALKBH5: AlkB homolog 5
ALYREF: Aly/REF export factor
AML: Acute myeloid leukemia
APC: Antigen presenting cells
APOE: Apolipoprotein E
BCAA: Branched-chain amino acid
BCAT1: Branched-chain amino acid transaminase 1
BMDMs: Bone marrow-derived macrophages
CAFs: Cancer-associated fibroblasts
CBC: Cap-binding complex
CDKN1A: Cyclin-dependent kinase inhibitor 1A
CESC: Cervical squamous cell carcinoma
CRC: Colorectal cancer
CSF-1: Cytokine macrophage colony-stimulating factor
CTLs: Cytotoxic T lymphocytes
DKK1: Dickkopf-related protein 1
DLBCL: Diffuse large B-cell lymphoma
DNMT2: DNA methyltransferase 2
EBV: Epstein–Barr virus
EC: Esophageal cancer
eIF3: Eukaryotic translation initiation factor 3
ELAVL1: Embryonic Lethal Abnormal Vision Like 1
FTO: Obesity-associated protein
GBM: Glioblastoma
GC: Gastric cancer
GLS: Glutaminase
Gys2: The liver-specific glycogen synthase
HBXIP: HBx-interacting protein
HCC: Hepatocellular carcinoma
HDGF: Heparin Binding Growth Factor
HIF: Hypoxia-inducible factors
hm5C: 5-Hydroxymethylcytidine
ICC: Intrahepatic cholangiocarcinoma
ICIs: Immune checkpoint inhibitors
ICOS: Inducible co-stimulatory
IFN: Interferon
IGF2BP1: Insulin-like growth factor 2 mRNA-binding protein 1
IL-12: Interleukin-12
LSCs: Leukemia stem cells
m1A: N1-methyladenosine
m3C: 3-Methylcytidine
m5C: 5-Methylcytosine
m6A: N6-methyladenosine
m7G: N7-methylguanosine
MAPK: Mitogen activated protein kinase
MDSCs: Myeloid-derived suppressor cells
METTL3: Methyltransferase-like 3
MM: Multiple myeloma
M-MDSCs: Monocyte-related myeloid-derived suppressor cells
MPNSTs: Malignant peripheral nerve sheath tumors
MTC: Methyltransferase complex
NAFLD: Nonalcoholic fatty liver disease
NML: Nucleomethylin
NSCLC: Non-small cell lung cancer
OXPHOS: Oxidative phosphorylation
PABP: Poly(A) binding protein
PCa: Prostate cancer
PDAC: Pancreatic ductal adenocarcinoma
PRMT1: Protein arginine N-methyltransferases 1
R255: Arginine 255
RAM: RNMT-activating miniprotein
RCC: Renal cell carcinoma
RCC2: Regulator of chromosome condensation 2
RNMT: RNA guanine-7 methyltransferase
SCD1: Stearoyl-CoA desaturase1
SCLC: Small-cell lung cancer
SHMT2: Serine hydroxymethyltransferase 2
SLE: Systemic lupus erythematosus
TAMs: Tumor-associated macrophages
TET1/2/3: Ten-eleven translocation proteins1/2/3
TIME: Tumor immune microenvironment
TME: Tumor microenvironment
TREX2: Three prime repair exonuclease 2
TRM61: TRNA methyltransferase catalytic subunit 61
TRMT10A: TRNA methyltransferase 10 homologue A
TRMT112: TRNA methyltransferase activator subunit 11–2
WBSCR22: Williams–Beuren syndrome chromosome region 22
WDR4: WD repeat domain 4
YBX1: Y-box-binding protein 1
YTHDF1: YTH N6-methyladenosine RNA binding protein 1
